# Ageing deformation of tailings dams in seasonally frozen soil areas under freeze-thaw cycles

**DOI:** 10.1038/s41598-019-51449-6

**Published:** 2019-10-21

**Authors:** Jiaxu Jin, Shiwang Li, Chenguang Song, Xinlei Zhang, Xiangfeng Lv

**Affiliations:** 1 Department of Civil Engineering, Liaoning Technical University, Fuxin, 123000 China; 2 School of Civil and Resource Engineering, University of Science and Technology Beijing, Beijing, 100083 China

**Keywords:** Environmental impact, Natural hazards

## Abstract

The freeze-thaw cycle is one of the important factors in inducing a dam-break in the permafrost region, so it is of great practical significance to study the mechanism of the failure deformation of tailings dams under freeze-thaw cycles. In this paper, the water-heat-force coupling model of a tailings dam considering frost-thaw damage is established, and the freeze-thaw cyclic ageing deformation of a tailings dam in a seasonally frozen soil area is studied. The correctness of the model is validated by numerical calculation. The research shows under the same water content, the compressive strength and modulus of deformation decrease with an increase in the number of freeze-thaw cycles, the cohesion and internal friction angle decrease, and the amplitude gradually decreases before becoming stable. In the process of cooling, the pore water pressure first increases and then decreases, and the pore water pressure first decreases and then increases during the heating process. The research results can provide a theoretical basis and reference values for the stability analysis of tailings dams in seasonally frozen soil areas.

## Introduction

The slope of a tailings dam in a seasonally frozen soil area is affected by natural freezing and thawing, which damages the structure of the tailings sand and decays the mechanical properties^1,2^. Water field redistribution in freeze-thaw tailings grains and accumulation of meltwater at the interface of freezing-thawing are very common^3–5^ and have resulted in widespread dam-breaking events in permafrost regions^6–8^. These events cause great harm to the national economy and the residents’ safety. The stability evaluation of the tailings dam in cold areas and the prevention and management of freeze-thaw disasters are key scientific problems^9,10^. It is of great theoretical and engineering significance to study the ageing deformation of tailings dams in seasonal permafrost areas under the action of freeze-thaw cycles, which is the basic mechanical problem directly facing the construction of tailings reservoirs in cold areas. In recent years, both domestic and international scholars have carried out some experimental and theoretical studies on the mechanical properties of tailings sand under the action of freeze-thaw cycles^11–14^. Through the undrained shear test of tailings sand after freeze-thaw cycles, Beier and Sego^15^ found that freeze-thaw cycles have a certain effect on improving the strength and surface stability of tailings sand. By carrying out the freeze-thaw cycles and a consolidation test of fine tailings, the effect of the pore ratio on the effective stress and permeability coefficient of tailings specimen was studied under the action of freeze-thaw cycles by Proskin *et al*.^16^. Yang^17^ used the artificial freezing method to study and analyse the physical and mechanical properties of tailings and their influencing factors under the condition of direct freezing and thawing. They also studied the effects of the freeze-thaw cycle on the permeability, consolidation compressibility and shear strength of the tailings via an indoor osmotic test, compression test and triaxial shear Test (UU). By setting up a separate open and sealed test environment, the environmental response of the specimen to the surface crack, water content, shear strength, cohesion and internal friction angle after different freeze-thaw cycles was studied by Ai *et al*.^18^, and the response of the mechanical properties to the environment was obtained via the mathematical fitting function. These studies mostly involve the change law of the basic mechanical characteristics of tailings sand, but there is less research on the deformation and internal force evolution law of tailings dams under the freeze-thaw cycles. The mechanism is not quite clear, and the deformation and internal force evolution of the tailings dam directly affects the stability of the tailings dam body. It is therefore very important to study the ageing deformation of tailings dam based on freeze-thaw cycles.

In this paper, the deformation mechanism and evolution of a tailings dam in Liaoning, China is studied by testing mechanical properties of the tailings sand and the growth model of the tailings sand under the action of freeze-thaw cycles. The research results can provide a basis for the safe operation of a tailings dam in seasonally frozen soil areas.

## Experimental Study on the Mechanical Properties of Tailings Sands under Freeze-Thaw Cycles

### Test materials

**Figure 1:**
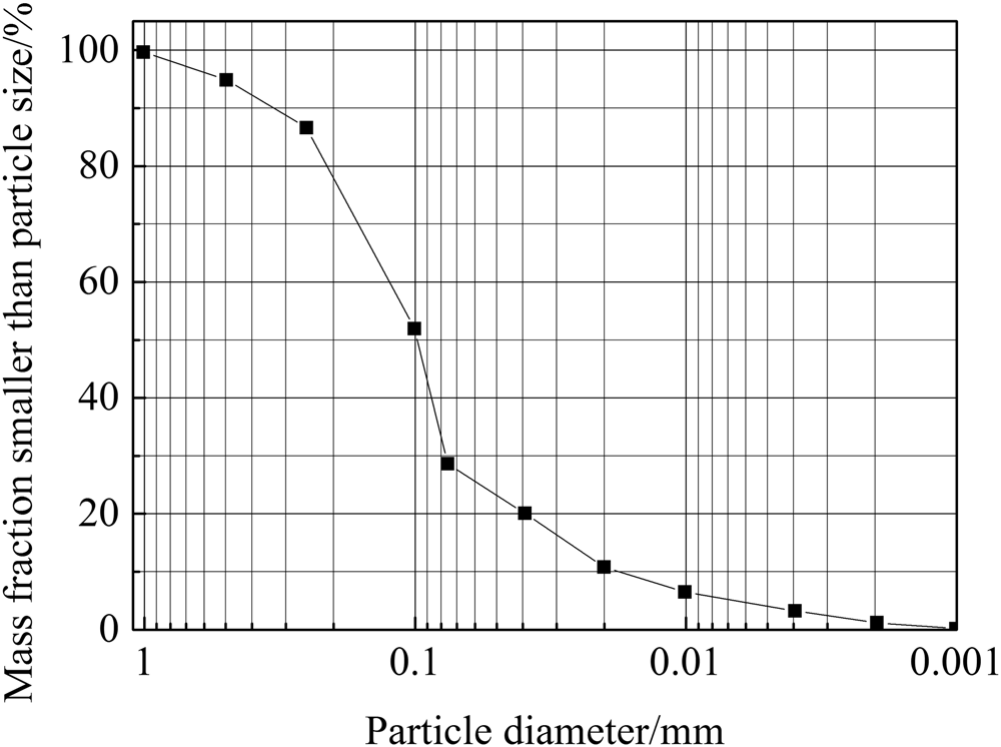
Particle size distribution (specific grading) curve.

The tailings sand used in the test was taken from the tailings reservoir discharge port. The sand was grey and black without any crushing or sieving treatment. A geotechnical test of the tailings sand was carried out according to the standard of the geotechnical test method. Figure 1 displays the specific grading curve. The main physical and mechanical indexes are shown in Table 1.

**Table 1:** Main physical and mechanical indexes of the tailings sand.

Specific gravity	Dry density /g·cm^−3^	Porosity ratio	Plastic limit/%	Liquid limit /%	Plastic Index	Permeability coefficient/cm·s^−1^	Compression factor/MPa^−1^	Modulus of compression /MPa
2.66	1.43	0.85	14.55	26.18	11.63	4.65 × 10^−4^	0.095	18.143

### Test equipment

**Figure 2:**
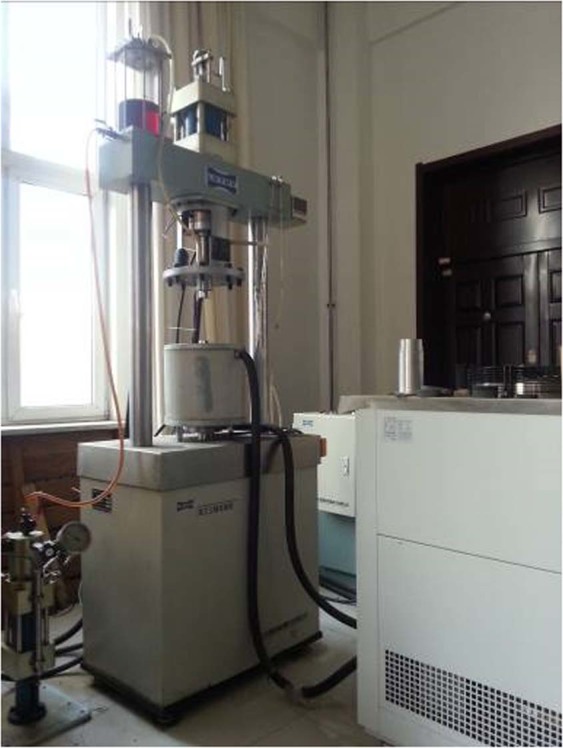
Permafrost triaxial testing machine.

In this test, the triaxial test equipment uses a frozen soil triaxial test machine (Fig. 2). The measuring range of the displacement sensor is 0–25 mm, the measuring accuracy is 0.03 mm, the confining pressure of the triaxial pressure chamber is 0–35 MPa, which is continuously adjustable, and the measuring accuracy of the confining pressure of the triaxial pressure chamber is +0.5% full scale. The freezing-thawing circulation equipment uses a high and low temperature test chamber. The measuring range of the temperature of the device is −40–150 °C, the measuring accuracy is 0.1 °C, the time can be set by itself, and the time range is 1–9999 hours.

### Experimental scheme

#### Experimental study on the mechanical properties of tailings sands under the action of freeze-thaw cycles

The factors affecting the strength of the tailings sand are dry density, water content, the freezing temperature, and the number of freeze-thaw cycles. The water content and number of freeze-thaw cycles were selected as the main controlling factors^19–21^, and the influence of the water content and the number of freeze-thaw cycles on the physical and mechanical properties of the tailings sands was studied by comparing the physical and mechanical properties of the tailings before and after the freeze-thaw cycles.

The sample size is 50 mm (diameter) × 80 mm (height), the freezing temperature is controlled at −20 °C, the melting temperature is controlled at 20 °C, and the freezing and thawing cycle time is 12 hours using the GDH-2005b type high and low temperature test chamber. Considering the effect of the water content on the physical and mechanical properties of the tailings sand, the water contents of the tailings sand samples are 11%, 13%, 15%, 17% and 19%. The maximum number of freeze-thaw cycles is set at 9^22,23^.

By measuring the height and moisture content of the samples before and after freezing and thawing, the water transfer law in the tailings sand under freeze-thaw action was studied. The test tailing is the same as in Section 3.1 mentioned above, and the initial moisture content of the test block is set at 19%. In the test, the tailings test block is sealed with a membrane to ensure that no water will evaporate. The influence of the freeze-thaw cycles on the height and internal moisture content of the sample is quantitatively analysed.

#### Model test of pore pressure growth of tailings sand under the action of freeze-thaw cycles

**Figure 3:**
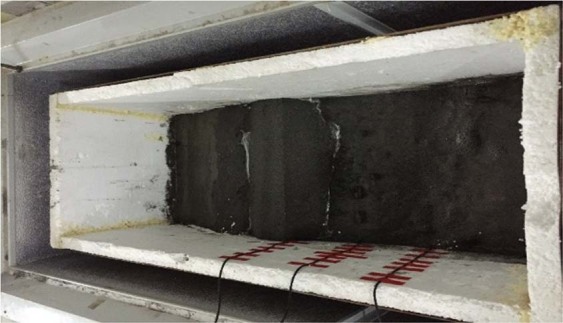
Pile-up process of tailings dam model.

**Figure 4:**
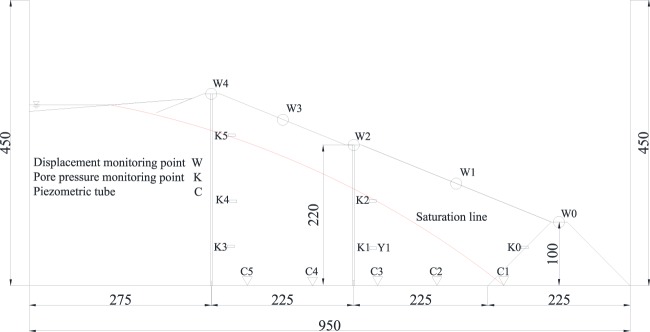
Schematic diagram of geometric model.

The size of the test model box is 950 mm × 300 mm × 450 mm (length × width × height). The model size is 950 mm × 300 mm × 300 mm. A total of 6 pore water pressure sensors are used in the test model, and the model box after stacking is shown in Fig. 3. The schematic diagram of geometric model is shown in Fig. 4. Putting the test model box in the high and low temperature test chamber, the freezing temperature is controlled at −20 °C, the melting temperature is controlled at 20 °C^24,25^, and the freezing and thawing cycle time is 12 hours. The maximum number of freeze-thaw cycles was set at 9. The pore water pressure before and after the freeze-thaw cycles at each monitoring point of the tailings dam was obtained by data acquisition system, and the change rule was analysed.

### Test results analysis

#### Analysis of the change in the stress-strain relationship of tailings sand under the action of freeze-thaw cycles

**Figure 5:**
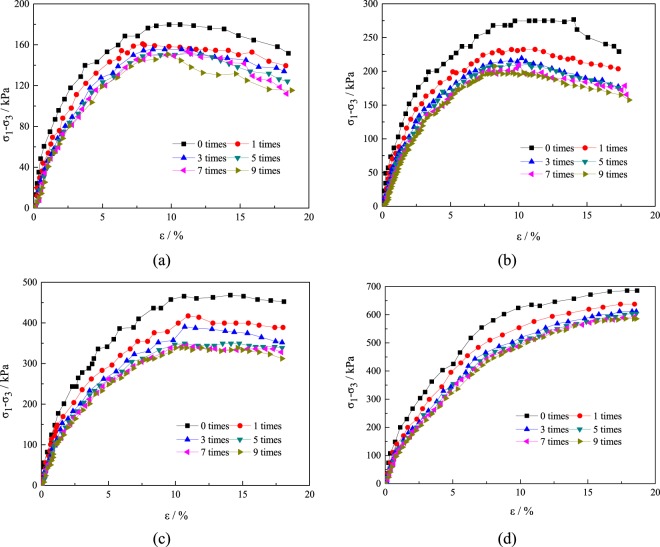
Relationship between deviatoric stress and strain under different confining pressures and freeze-thaw cycles.

The stress-strain curves of the prepared tailings sands (water content of 15%) were tested with different confining pressure (50 kPa, 100 kPa, 200 kPa and 300 kPa) and different freeze-thaw cycles (1, 3, 5, 7 and 9), with the changes in the stresses-strains shown in Fig. 5. In general, an increase in the confining pressure results in a gradual increase in the peak deviatoric stress of the tailings sand. When the confining pressure is 50 kPa and 100 kPa, the peak rear is obviously softened, and when the confining pressure rises to 200 kPa, the softening phenomenon and ductility phenomenon appear successively. When the confining pressure reaches 300 kPa, There is no peak, and the hardening characteristic is always present. At this time, 15% of the axial strain is taken as the peak deflection stress. Under the same confining pressure, the stress strain curve gradually moves down with an increase in the number of freeze-thaw cycles, and the amount of change gradually decreases. In particular, the decrease in the stress strain curve is the largest when the number of freeze-thaw cycles is 1, followed by 3 freeze-thaw cycles. When the number of freeze-thaw cycles is 5–9, the variation is not significant. It can be seen that the stress-strain curve of the specimen is not affected by the number of freeze-thaw cycles after the number of freeze-thaw cycles reaches 5.

**Figure 6:**
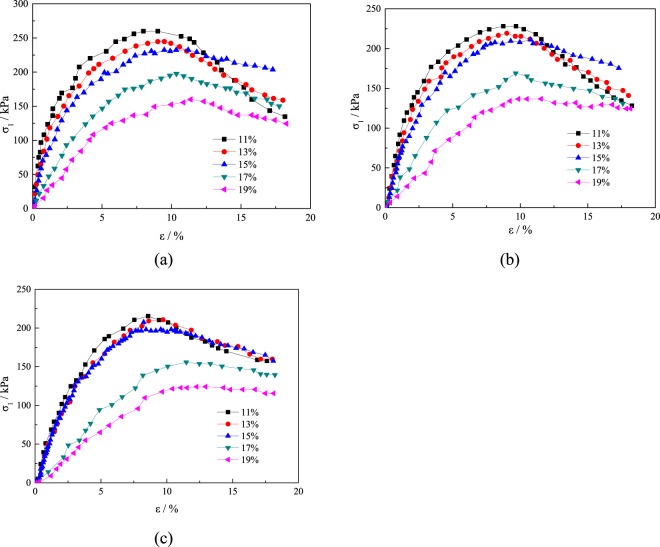
Stress-strain curves of tailings under different numbers of cycles of freeze-thaw.

The change in water content can change the structure of a solidified soil and make its mechanical properties change. Because the stress-strain relationship of tailings under different confining pressures is roughly the same, the stress-strain curves of the tailings with different moisture contents under 1, 5 and 9 freeze-thaw cycles at confining pressures of 100 kPa are listed, as shown in Fig. 6.

#### Analysis of the variation in compressive strength and deformation modulus of tailings sands under the action of freeze-thaw cycles

**Figure 7:**
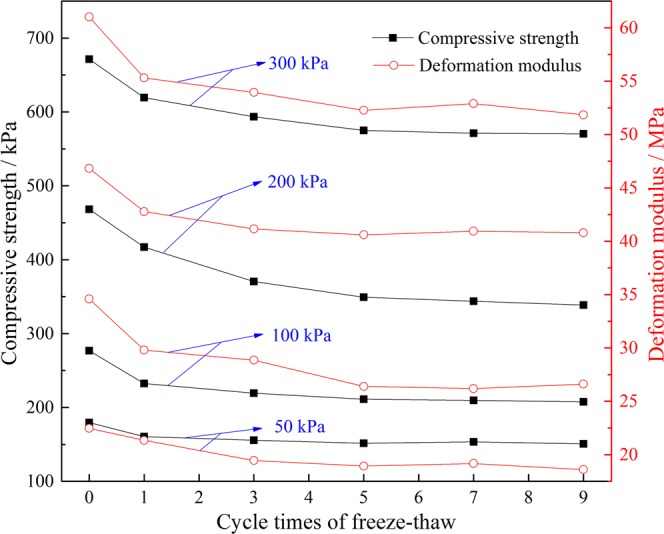
Curves of the number of freeze-thaw cycles with the compressive strength and the deformation modulus under different confining pressures.

Figure 7 is the relation curve between the peak stress and the deformation modulus and the number of freeze-thaw cycles under different confining pressure conditions. As seen from the figure, when confining pressure is 50 kPa, the change trend of the peak deviatoric stress under 9 freeze-thaw cycles is as follows: a decrease of 10.7%, 3%, and 2.6%, an increase of 1.2%, and a decrease of 1.7%. It can be seen that after the 5th freeze-thaw cycle, the effect of the number of freeze-thaw cycles on peak strength is not obvious and gradually becomes stable.

It can also be seen from the graph that the number of freeze-thaw cycles significantly affects the deformation modulus of the tailings sand, and the deformation modulus decreases gradually with an increase of the freeze-thaw cycles. After nine freeze-thaw cycles, the deformation modulus of tailings under 4 groups of confining pressures decreased by 23.9%, 25.5%, 12.8% and 15.1%, respectively. And the initial reduction degree is larger, the later reduction degree decreases gradually, before finally stabilizing. It can be seen that the deformation modulus after 5 freeze-thaw cycles under 4 groups of confining pressures reached the lowest value, after the 7th cycle there was a slight rebound, and then there were almost stable changes. Taking 50kPa confining pressure as an example, the deformation modulus of tailings sand decreased by 12.7%, 8.9%, 2.6%, −1.2% and 2.9% respectively after different freeze-thaw cycles (1, 3, 5, 7 and 9).

The main reasons for this phenomenon are as follows: in the early stage of freeze-thaw cycle (0–5 times), the ice body expands in the fine structure of tailings sand particles during low temperature freezing, then it melts after heating, and the repeated freezing-thawing-freezing action is characterized by ice crystallization and moisture migration in tailings sand, which causes irrecoverable damage to the structure of tailings particles. The strength decreases and the corresponding deformation modulus decreases at a higher rate. The “slight rebound” is because when the number of cycles reaches 6~7, the mechanical properties of the tailings sand (cohesion c, internal friction angle φ and deformation modulus e) have essentially stabilized, so the tailings sand particles transition from an unstable state in the previous 5 freeze-thaw cycles to a stable state in cycles 6–7, resulting in the overall skeleton stress increasing but with a low amplitude. In the later stage of freeze-thaw cycle (8–9 times), the stable structure just reached by tailings sand structure was destroyed again, but the damage rate of tailings sand by freeze-thaw was greatly reduced compared with the initial stage, which showed that the deformation modulus decreased again, and the reduction was small.

**Figure 8:**
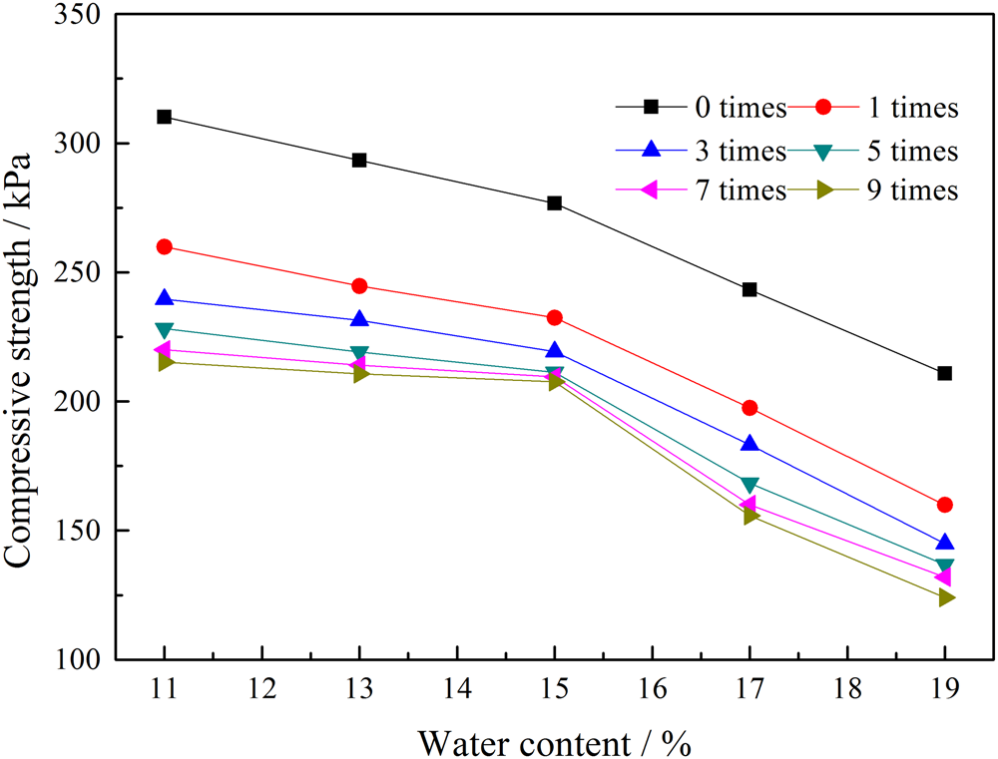
Curves of water content and compressive strength under different numbers of freeze-thaw cycles.

The compressive strength of the tailings sands with different freeze-thaw cycles at different water contents at 100 kPa is shown in Fig. 8. With an increase in the water content, the compressive strength of the tailings sands under different freeze-thaw cycles shows a decreasing trend with essentially the same change law. The main reason is that the increase in the water content in the tailings sand increases the pore water contents of the grains. Then, the ice body freezes, causing large expansion, and then melts after heating. This process is also accompanied by the precipitation and water migration^26^. The irreversible damage caused by the freeze-thaw-freeze effect on the tailings sand particles increases, and the compressive strength decreases. With an increasing number of freeze-thaw cycles, the influence of the water content on the compressive strength of the tailings sand increases, especially when the water content is above 15%, resulting in the amplitude of compressive strength increasing.

#### Analysis of the change in cohesion and internal friction angle under the action of freeze-thaw cycles

**Figure 9:**
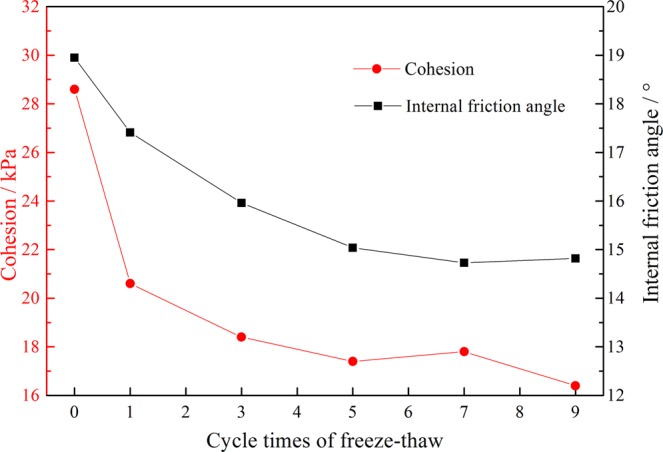
Curves on the cohesion and internal friction angle with different numbers of freeze-thaw cycles.

The changes in the cohesion and internal friction angle of tailings sand after different numbers of freeze-thaw cycles are shown in Fig. 9. With an increase in the number of freeze-thaw cycles, both the cohesion and internal friction angle showed a decreasing trend on the whole, stabilizing after the number of freeze-thaw cycles reached 5. The cohesion had a slight rebound in the 7th freeze-thaw cycle, which was consistent with the trend of the peak strength and deformation modulus; the internal friction angle rebounded slightly during the 9th freeze-thaw cycle. The change in cohesion from cycles 1 to 9 was reduced by 27.97%, 10.68%, and 5.43%, increased by 2.3%, and reduced by 2.3% from an initial 28.6 kPa to an eventual 17.4 kPa, with a reduction amplitude of 39.2%. The internal friction angle decreased by 8.12%, 8.3%, 5.8%, and 2.1%, and then increased by 0.6% from an initial 18.95 degrees to 14.82 degrees, reducing by 21.8%. Based on the data of cohesion and internal friction angle, the shear strength of tailings sand samples under different freeze-thaw cycles can be calculated. Taking 100 kPa vertical pressure as an example, the shear strength of tailings sand samples after different freeze-thaw cycles are 62.94 kPa, 51.96 kPa, 47.00 kPa, 44.27 kPa, 44.09 kPa and 42.86 kPa, respectively. The gradual decrease of shear strength verifies the effect of freeze-thaw damage.

When the tailings are frozen, the internal water freezes and expands. Additionally, the tailings sand particles are in cold shrinkage, so the connection between the particles are destroyed. When the temperature rises, the internal water melts, particles gradually expand, but the connection of the frozen damage cannot be restored. Repeated freeze-thaw cycles eventually lead to the tailings sand particle structure being gradually weakened, resulting in a decrease in cohesion. Thus, the shear strength of the tailings sand is reduced.

After several freeze-thaw cycles, the overall trend of the strength index of tailings is as follows: Cohesion c and internal friction angle φ decrease as a whole, but increase in a certain range, and their changes are not consistent in time. The main reasons are as follows: the cohesion of the tailings sand mainly comes from the interaction of electrostatic attraction, Waals, cementation force between particles, false cohesion (capillary), etc. The electrostatic molecular force depends mainly on the mineral composition and density, the density of tailings sand will be reduced after freezing and thawing, which will lead to a different degree of cohesion reduction. The freezing-thawing damage of the tailings sand structure also makes the cementation force decrease, which causes the cohesion to decrease. After the tailing sand is frozen and melted, the inner pores of tailings grains change, which causes a rearrangement of the tailings. Under high confining pressure, there has greater tightness. In addition, during the freezing process of tailings sand, the volume of water in the pores will increase after freezing, which causes the pore characteristics of tailings sand to clearly change, leading to more contact points between the tailings grains, which is beneficial for the increase in the friction angle. In addition, because cohesion c and internal friction angle φ are not absolutely positively correlated with shear strength, the cohesion and internal friction angle will increase in a certain range even if the shear strength of tailings sand sample decreases gradually under freeze-thaw cycles.

#### The change in pore water pressure of the tailings sand under the action of freeze-thaw cycles

**Figure 10:**
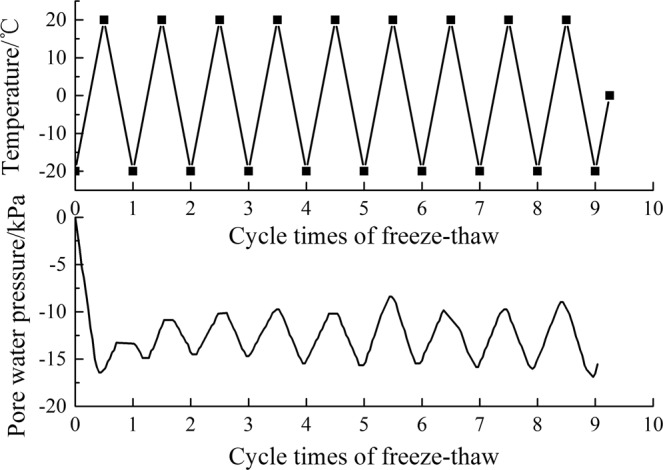
Change curves of the pore water pressure (K2) under the action of freeze-thaw cycles.

Under the action of freeze-thaw cycles, the pore water pressures at the 6 monitoring points are approximately the same. Taking the K2 monitoring point as an example, the variation in the pore water pressure with the temperature is shown in Fig. 10. It can be determined from the diagram that the pore water pressure begins to change periodically with temperature after 2 freeze-thaw cycles. Through an analysis of one cycle, it can be determined that the pore water pressure first increases and then decreases with a decrease in temperature during the cooling process. The pore water pressure first decreases and then increases with an increase in temperature during the heating process.

The reason for this change in the pore water pressure is that during the cooling process, when the temperature is higher than the freezing point, as the ice continues to melt into water, the water content in the tailings’ pores increases, as does the pore water pressure. When the temperature is lower than the freezing point, the water begins to turn into ice, and the capillary potential and the adsorption potential of the water in the tailings sand decrease. Thus, the pore water pressure is reduced. The capillary potential is mainly controlled by the ice water interface curvature radius; the smaller the curvature radius of the ice water interface, the smaller the capillary potential and the smaller the pore water pressure. The adsorption potential is mainly controlled by the thickness of the unfrozen water film; the smaller the thickness of the unfrozen water film, the smaller the adsorption potential and the smaller the pore water pressure. With a decrease in the temperature, the thickness of the unfrozen water film decreases, so the adsorption potential is smaller, and the pore water pressure decreases. To summarize, in the cooling process the pore water pressure first increases, then decreases. In the heating process, the pore water pressure first decreases, then increases^27^.

## Numerical Simulation

### Water-heat-force coupled model equation of a tailings dam considering frost-thaw damage

#### Basic equations

1. Basic equation of temperature field.
  If the effect of thermal convection is neglected, the heat conduction equation^28^ for phase transformation is:
  1 $$C\frac{\partial T}{\partial t}=div(\lambda gradT)+L{\rho }_{{\rm{i}}}\frac{\partial {\theta }_{{\rm{i}}}}{\partial t}$$
  An introduction to the concept of equivalent water content^29^ can be simplified to:
  2 $$\overline{C}\partial T/\partial t=div(\lambda gradT)+L{\rho }_{w}\partial {\theta }_{w}/\partial t$$
  where $\overline{C}=C+L{\rho }_{w}\partial {\theta }_{w}/\partial T$ is the equivalent heat capacity, ${\theta }_{{\rm{w}}}={\theta }_{u}+{\rho }_{i}{\theta }_{i}/{\rho }_{w}$, ${\theta }_{w}$ is the equivalent water content, *C* is the volume ratio of the tailings sand, *L* is the thermal capacity of the volume phase of ice water, *T* is the temperature, *W*_*u*_ is the volume content of the unfrozen water, *W*_*i*_ is the volume content of the ice, ${\rho }_{i}$ is the weight of the ice, and ${\rho }_{w}$ is the water bulk density.
2. Basic equation of seepage field.
  Considering the influence of the ice water phase transformation and tailings sand strain, the diffusion equation of water transfer in the tailings sand^30^ has the following expression:
  3 $$\frac{\partial {\theta }_{u}}{\partial t}=\frac{\partial }{\partial x}(D({\theta }_{u})\frac{\partial {\theta }_{u}}{\partial x})+\frac{\partial }{\partial y}(D({\theta }_{u})\frac{\partial {\theta }_{u}}{\partial y})-\frac{{\rho }_{i}}{{\rho }_{w}}\frac{\partial {\theta }_{i}}{\partial t}+\frac{\partial k({\theta }_{u})}{\partial y}+\frac{\partial \varepsilon }{\partial t}$$
  If the equivalent water content ${\theta }_{w}$ is introduced, the above equation can be obtained:
  4 $$\frac{\partial {\theta }_{w}}{\partial t}=\frac{\partial }{\partial x}(D({\theta }_{u})\frac{\partial {\theta }_{u}}{\partial x})+\frac{\partial }{\partial y}(D({\theta }_{u})\frac{\partial {\theta }_{u}}{\partial y})+\frac{\partial k({\theta }_{u})}{\partial z}+\frac{\partial \varepsilon }{\partial t}$$
  where $D({\theta }_{u})$ is the water diffusion rate of unfrozen water, and $k({\theta }_{u})$ is the water conductivity of tailings sand.
3. Basic equation of stress field.

In saturated conditions, there is a linear relationship between the pore ratio e and the effective stress $\sigma ^{\prime}$ of the tailings sand^31^, namely:

5 $$e=e-{\alpha }^{\ast }\sigma ^{\prime} $$

where ${e}_{0}$ is the initial porosity ratio of tailings sand, and ${\alpha }^{\ast }$ is the compaction coefficient of tailings.

During the process of tailing sand freezing, the volume change in the positive-frozen tailings sand is influenced by two factors: the water phase becoming ice and water migration^32^. The volume expansion strain^33,34^ of the tailings sand can be expressed as:

6 $${\varepsilon }_{v}={\varepsilon }_{vf}+{\varepsilon }_{vT}$$

where ${\varepsilon }_{vf}$ is the volumetric strain caused by the phase change of ice water in tailings sand, and ${\varepsilon }_{vT}$ is the volumetric strain caused by the temperature change.

#### Equation of energy balance

The differential form of the energy balance equation is expressed as:

7 $$-{q}_{i,i}+{q}_{v}=\frac{\partial \zeta }{\partial t}$$

where ${q}_{i}$ is the heat flux vector, with units of W*/*m^2^, ${q}_{v}$ is the volume heat source intensity, with units of W/m^3^, and ζ is the unit volume storage heat, with units of J/m^3^. Usually, the change in energy storage and the volumetric strain may cause a temperature change, and the thermal constitutive relation of the correlation parameter is:

8 $$\frac{\partial T}{\partial t}={M}_{th}(\frac{\partial \zeta }{\partial t}-{\beta }_{th}\frac{\partial \varepsilon }{\partial t})$$

where *M*_*th*_ and *β*_*th*_ are the material constant and *T* represents the temperature. Here, specific conditions are considered: *β*_*th*_ = 0, and ${\beta }_{th}=\frac{1}{\rho {C}_{v}}$. *ρ* is the mass density of the medium, with units of kg/m^3^, and *C*_*v*_ is the specific heat of the fixed volume, with units of J/kg °C. It is assumed that the effect of the strain change on the temperature is approximately ignored, and the hypothesis is effective for the quasi-static problem of the solids and liquids involved. Therefore, to the following is available:

9 $$\frac{\partial \zeta }{\partial t}=\rho {C}_{v}\frac{\partial T}{\partial t}$$

The following energy balance equation is obtained by substituting (9) into (7):

10 $$-{q}_{i,i}+{q}_{v}=\rho {C}_{v}\frac{\partial T}{\partial t}$$

#### Convection heat diffusion conduction energy equilibrium equation

11 $${c}^{T}\frac{\partial T}{\partial t}+\nabla \cdot {q}^{T}+{\rho }_{0}{c}_{w}{q}_{w}\cdot \nabla T-{q}_{v}^{T}=0$$

where *T* is the temperature, *q*^*T*^ is the heat flux, ${q}_{w}$ is the flow rate for the fluid, ${q}_{v}^{T}$ is the volume heat source intensity, ${\rho }_{0}$ and ${c}_{w}$ are the reference fluid density and specific heat, respectively, and ${c}^{T}$ is the effective specific heat.

#### Equation of heat conduction equilibrium

12 $${q}^{T}=-{k}^{T}\nabla T$$

where *k*^*T*^ is an effective heat conduction coefficient, which is isotropic in the advection equation, and the effective heat conductivity is defined by the thermal conductivity of solids and liquids. The relationship between ${k}_{s}^{T}$ and ${k}_{w}^{T}$ is as follows:

13 $${k}^{T}={k}_{s}^{T}+nS{k}_{w}^{T}$$

#### Boundary conditions

The temperature boundary condition is: ${T|}_{s}={T}_{b}$, or ${\frac{\partial T}{\partial n}|}_{s}={T}_{b}$, or ${(\frac{\partial T}{\partial n}+T)|}_{s}={T}_{b}$

The initial conditions of temperature field are: $T{|}_{t=0}={T}_{0}$

where *s* is a boundary, *n* is the outer normal direction of the boundary, *T*_*b*_ is a boundary temperature, or a boundary temperature gradient, and *T*_0_ is the initial temperature distribution.

### Design of numerical calculation scheme

**Figure 11:**
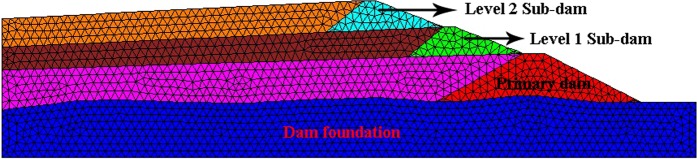
Numerical model diagram.

The water-heat-force coupled model of the tailings dam considering frost-thaw damage is introduced into the numerical calculation. The correctness of the mathematical model is validated by comparing the calculated results with the experimental results of the pore water pressure at the same monitoring point. The size and material parameters of the numerical model are in accordance with the actual model test conditions, and the specific calculation parameters are shown in Table 2. The geometric model shown in Fig. 4 is imported into the Ansys grid, then imported into flac3d, generating 6,087 nodes and 25,691 units, as shown in Fig. 11. In the experiment, the model shown in Fig. 11 was subjected to nine freeze-thaw cycles, in which the freezing temperature was −20 °C, the thawing temperature was 20 °C, and the freezing and thawing time for one cycle was 12 hours.

**Table 2:** Model parameter table.

Materials	Density/kgm^−3^	Cohesion/kPa	Internal friction angle/°	Modulus of elasticity/MPa	Poisson’s ratio	Shear Modulus/MPa
Primary Dam	1780	20	28	201.354	0.41	160.0
Sub-dam	1750	10	30	205.279	0.41	170.0
Tailings sand	1710	10	36	124.576	0.41	44.1
Dam foundation	2400	400	38	500.237	0.38	196.6

### Analysis of numerical results

**Figure 12:**
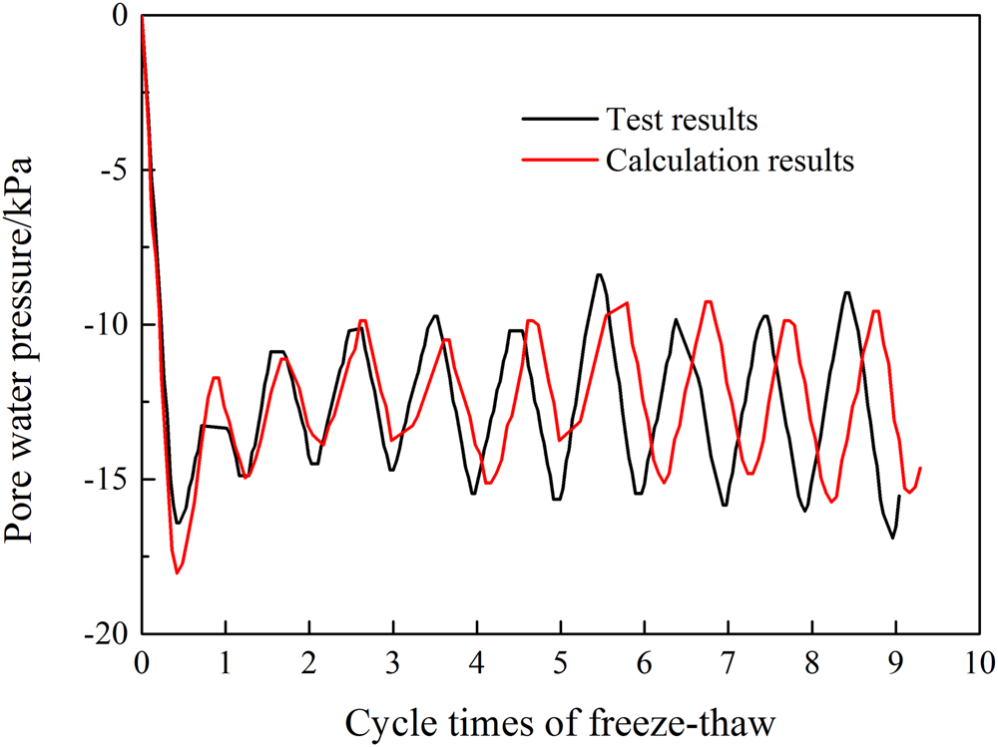
Comparison of the experimental and numerical results of the pore water pressure.

Figure 12 is the comparison curve between the calculated results of the pore water pressure and the experimental results. It can be seen from the graph that the calculated results are in good agreement with the test results, and the numerical values are consistent. From the comparison of the above two methods, the change law of the calculated pore water pressure is correct and can be used in subsequent calculations.

## Discussion

When the tailings sand samples change periodically at positive and negative temperatures, the moisture in the samples changes continuously. The volume expansion of water in the sample pore is about 9% after solidification, and the resulting expansion force will squeeze tailings particles, resulting in the destruction of cementation between aggregates and aggregates, and between aggregates and minerals. The pore between aggregates is gradually expanded, irreversible deformation occurs, the structure of the sample is destroyed, the pore size increases as a whole, the internal structure of the sample is loose, and the macro-mechanical strength decreases. Moreover, repeated freezing and thawing makes the tailings develop from unstable state to new dynamic stable state. Thus, in the repeated freeze-thaw cycles, the damage of the pore structure caused by frost heave is the reason for the decrease of macro-mechanical strength. Therefore, in order to find out the effect of freeze-thaw cycles on the pore structure of tailings and reveal the mechanism of damage of freeze-thaw cycles on the structure of tailings samples, the meso-pore structure test of freeze-thaw tailings will be carried out in the future, including SEM test and MIP test.

## Conclusions

1. Under the same confining pressure, the stress strain curve gradually moves down with an increase in the number of freeze-thaw cycles, and the amount of change gradually decreases. The stress-strain curve of the specimen is not affected by the number of freeze-thaw cycles after the number of freeze-thaw cycles reaches 5.
2. With the same moisture content, the compressive strength and modulus of deformation decrease with an increase in the number of freeze-thaw cycles. The cohesion and internal friction angle decrease, and the amplitude decreases gradually before finally becoming stable. After 9 freeze-thaw cycles, the cohesion decreased to 17.4 kPa, decreasing the amplitude by 39.2%. The internal friction angle was reduced to 14.82°, decreasing by 21.8%.
3. Under the action of freeze-thaw cycle, cohesion c and internal friction angle φ decrease as a whole, but increase in a certain range, and their changes are not consistent in time.
4. In the process of cooling, the pore water pressure first increases and then decreases with the temperature decreasing, and the pore water pressure first decreases and then increases with an increase in temperature during the heating process.

### Statistical analysis

Statistical signifcance was evaluated with an unpaired, two-tailed Student’s T test. A *p* value < 0.05 was considered signifcant. Unless otherwise noted, all statistic data shown are the means ± S.D. in triplicate cultures. When representative images were shown, they represent at least three samples.

## Data Availability

All data supporting the findings in this study are available from the corresponding author on reasonable request.
